# Effects of the Temperature and Salt Concentration on the Structural Characteristics of the Protein (PDB Code 1BBL)

**DOI:** 10.3390/polym14112134

**Published:** 2022-05-24

**Authors:** Dongqing Shao, Qun Zhang, Peng Xu, Zhouting Jiang

**Affiliations:** Department of Applied Physics, China Jiliang University, No. 258 Xueyuan Street, Xiasha Higher Education Zone, Hangzhou 310018, China; dongqing1756@163.com (D.S.); zhangqun19970217@163.com (Q.Z.); xupeng@cjlu.edu.cn (P.X.)

**Keywords:** molecular dynamics simulation, protein molecules, salt solution, temperature

## Abstract

The effect of the temperature and salt solution on the structural characteristics of the protein 1BBL was investigated by molecular dynamics simulations. The paper presents simulation results regarding the non-bonded energy and the structural stability of the protein immersed in salt solutions with different concentrations and temperatures. Our work demonstrates that the electrostatic potential energy and van der Waals energy of the system show the opposite changes with the influence of the external environment. Since the electrostatic potential energy changes more obviously, it is dominated in the non-bonding interactions. The structural parameters, such as the root mean square deviation and the radius of gyration, increased initially and decreased afterward with the increase of the salt concentration. The protein presented the loose structure with a relative low stability when it was immersed in a monovalent solution with a salt concentration of 0.8 mol/L. The salt concentration corresponding to the maximum value of structural parameters in the monovalent salt solution was double that in the divalent salt solution. It was also concluded that the protein presented a compact and stable structure when immersed in salt solutions with a high concentration of 2.3 mol/L. The analysis of the root mean square deviation and root mean square fluctuation of the protein sample also exhibited that the structural stability and chain flexibility are strongly guided by the effect of the temperature. These conclusions help us to understand the structural characteristics of the protein immersed in the salt solutions with different concentrations and temperatures.

## 1. Introduction

A protein composed of sequences of amino acids has a unique (native) three-dimensional structure in the natural environment. The biological function of a protein strongly depends on its unique structure, determined by complicated interactions among residues. The prediction of the native three-dimensional structures of proteins from the knowledge of their sequence of amino acids can be achieved according to the global minimum on the free-energy landscape of a protein [1]. However, in the presence of external factors, such as ionic strength, PH value, temperature, electric/electromagnetic fields, and a crowded cellular environment, the misfolding and aggregation of non-native protein structures are observed [2,3,4]. One way to investigate the effects of nonspecific interactions, which profoundly affect the behaviour and structural stability of the protein, is to measure the translational diffusion coefficients, second virial coefficients, and scattering intensities of protein solutions [5,6,7]. Although these methods could partly give more information about the protein in the solvent, difficulties are usually encountered due to the limitation of measurement techniques [8,9]. With the rapid development of computer technology, computational biophysics has started to focus on researching the structure and interactions of biomolecules at the atomistic level. It has become another powerful method to discover the inherent mechanisms of molecular biology [10,11,12,13].

A solvent medium is critical in controlling the structure and dynamics of a protein and to the performance of its specific function. How the solvent affects the properties of a protein depends on many variables, such as the temperature [14], ion type [15], electric field [16], or protein or solvent concentration, etc [17]. Temperature is a significant variable parameter, as proteins respond differently in high and low temperature conditions. Peng Sang et al. have performed comparative molecular dynamics simulations on thermophilic serine protease (THM) and its homologous mesophilic counterpart (PRK). They concluded that the solvent (entropy force) plays a significant role in protein adaption at high temperatures [18]. Many proteins have a high thermal stability, while others can unfold or even denature at high temperatures [19,20,21]. To understand the structural properties of proteins, it is also necessary to study the possible conformational changes that could arise when the proteins are subjected to a temperature condition. Intrinsically disordered proteins have been reported to display temperature-induced partial folding or secondary structure formation [22]. Computational methods to predict the *T*_m_ (midpoint of temperature-induced protein unfolding curves) of proteins have also been proposed [23]. Moreover, salts affect widely different properties of proteins, such as their stability, solubility, and biological function [24]. Miao et al. presented the local movement of residues and subsequent structures, depending on the combined action of the residues type, solvent concentration, and temperature [14]. Arakawa et al. exerted specific effects on proteins, which depend on the nature of the salt and its concentration, resulting in either the stabilization or denaturation of proteins, as well as in their salting-in or salting-out [25]. At low ion concentrations, protein surfaces are charged, preventing aggregation and increasing protein solubility [26,27]. With the increase of ionic strength, proteins are salting out in protein aggregation and precipitation [28]. This research provides fundamental information on the temperature- and salt-dependence of protein dynamics. However, the thermal stability of natural proteins is still particularly challenging, as it is generally limited to a narrow range of temperatures, outside of which proteins frequently denature with a concomitant loss of function. The interplay between the cooperative and competing effect of residue–solvent and residue–residue interactions and temperature on the covalently bonded residues in a protein, which leads to the interesting structural and dynamical response, is still unclear.

Globular proteins are categorized into four structural classes, namely all-α, all-β, α+β, and α/β proteins, based on the topology and content of its secondary structure, i.e., α-helices and β-sheets. The previous research was mainly focused on the proteins classified as α+β and α/β types, with both an α-helix and β-sheet on its secondary structural level. To give a contribution in comprehending the conformational transition, especially the evolutions of α-helical structures of the protein, we have selected dihydrolipoamide dehydrogenase (PDB code: 1BBL), classified into all-α proteins, which comprises of two α-helix fragments without a β-sheet in its initial conformation, as a study case [29]. In this article, we used MD simulation to explore the effect of thermal and solvent interaction, including NaCl, KCl, CaCl_2_, and MgCl_2_ with different concentrations, on the protein 1BBL. This work is organized as follows: following the introduction in Section 1, the methodology, including the construction of the simulation box and molecular dynamics simulation details, are presented in Section 2. The simulation results regarding the effects of the temperature, the type of ions, and salt concentration are discussed in Section 3. Section 4 contains the concluding remarks of this study.

## 2. Simulation Details and Methods

The E3-binding domain of the dihydrolipoamide in the 2-oxoglutarate dehydrogenase (2-OGDH) complex of *Escherichia coli* was investigated as a protein sample in the present work. The initial configuration of the objective protein (PDB code: 1BBL) was obtained from the Protein Database Bank (PDB). The protein sample, classified as an all-α protein by the Structural Classification of Proteins (SCOP) database, comprises two alpha-helix segments (No. 14–23 residues and No. 41–47 residues). The protein 1BBL, made up of just 37 amino acids, has a net positive charge. The chloride ions were added to the system to neutralize it.

All simulations were performed by molecular dynamic algorithms implemented in the NAMD 2.6 software package (Beckman Institute, University of Illinois at Urbana-Champaign, Urbana troop, IL, USA) using an all-atom CHARMM27 force field [30]. The protein configuration was enclosed in the center of the periodic cubic simulation box. The size of the simulation box was about 1.3 × 1.3 × 1.3 nm^3^. The periodic boundary conditions were applied so that edge effects were avoided and the systems behaved like bulk solutions. Four kinds of salt solutions, i.e., CaCl_2_, MgCl_2_, NaCl, and KCl with different concentrations and temperatures, were selected as external solvent conditions. The water molecules were modelled with the TIP3P model. The protein sample 1BBL was immersed into the salt solution with a concentration from 0.1 mol/L to 2.3 mol/L, respectively. The range of temperature was set from 280 K to 390 K. First, the simulation system was energy minimized under the convergence criterion of the maximum force value of 10 kJ/nm/mol by the steepest descent method for 60,000 steps. Then, the molecular dynamics simulations were carried out for 10 ns under the condition of a certain temperature and constant pressure P = 1 atm. The time step and mesh spacing were set as 2 fs and 0.1 nm, respectively. The van der Waals interactions were calculated by the switching function, which started at a distance of 1.0 nm and reached zero at 1.2 nm. The long-range electrostatic interactions were calculated by the particle grid Ewald (PME) method. The simulated system interacted via the non-bonded potentials and bonded potentials, including bond-stretching, bond-bending, and bond-torsional potentials. In this article, non-bonded energies, including electrostatic energy and van der Waals energy, are mainly discussed, since the bonded interactions have a relatively low relevance with the salt solution.

In this study, we carried out MD simulations to explore the effects of the salt solution and temperature on the structural characteristics of the protein 1BBL. The radius of gyration, *Rg,* is a basic measurement of the overall size of a chain molecule. The change in the structure of a protein during MD simulations can be quantified by the radius of gyration. *Rg*, which is defined as:

(1) $$R_{g}=\sqrt{\frac{1}{N}\sum_{i=1}^{N}{\left|r(i)-r_{center}\right|}^{2}}$$

where *N* is the number of protein atoms. *r*(*i*) and *r_center_* are the coordinates of an atom *i* and the center of mass, respectively. The conformational stability of proteins during the simulation procedure was examined by calculating the root mean square deviation (*RMSD*) and root mean square fluctuation (*RMSF*). The *RMSD* is a numerical measurement of the conformational changes between two structures. It is defined as:

(2) $$RMSD=\sqrt{\frac{1}{N}\sum_{i=1}^{N}{\left|r_{final}(i)-r_{initial}(i)\right|}^{2}}$$

where *N* is the number of protein atoms. *r_final_*(*i*) and *r_initial_*(*i*) are the coordinates of an atom *i* in its final structure and initial structure, respectively. The *RMSF* represents the fluctuation of the coordinates of each atom of the protein near its reference coordinate during the simulation process, and it is an important tool to characterize the freedom of the center $C^{\alpha }$ atoms in protein molecules, which is defined as:

(3) $$RMSF_{i}=\sqrt{\frac{1}{t_{total}}\sum_{t_{j}=1}^{t_{total}}{\left|r_{i}(t_{j})-r_{i}^{ref}\right|}^{2}}$$

where *t_total_* is the total simulation time, and the reference coordinate, *r^ref^*, is the average coordinate of the $C^{\alpha }$ atom during the whole simulation period.

## 3. Results and Discussion

### 3.1. Effect of the Salt Solution

#### 3.1.1. Energy Analysis

**Figure 1:**
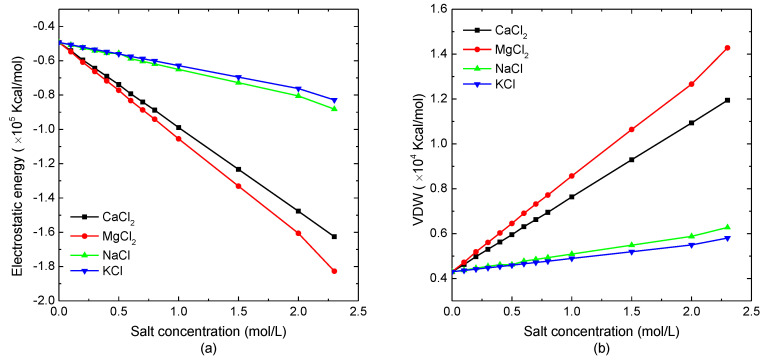
The non-bonded interactions of the protein system versus the external salt concentration. (**a**) Electrostatic potential energy and (**b**) van der Waals energy.

The salt concentration dependence of non-bonded interactions of a single protein molecular was investigated. As the bond-stretching, bond-bending, and bond-torsional potentials are almost the same in different kinds of salt solutions with various concentrations, the electrostatic potential energy and van der Waals energy of the model system are mainly discussed in this article. The simulation results of the electrostatic energy and van der Waals energy of the protein system versus salt concentration are shown in Figure 1a,b, respectively. As demonstrated in Figure 1a, the electrostatic energy decreased linearly with the increase of the salt concentration. Comparing the protein sample immersed in different salt solutions, the electrostatic energy of the simulated system in the divalent ion solution decreased more violently than in the monovalent ion solution. The analysis of the data shown in Figure 1a could conclude that the declining slope of electrostatic energy, as the function of divalent ion salt concentration, was about four-times that of the protein in the monovalent salt solution. It indicated that the increase of the total number of charged ions caused the decrease of the electrostatic potential energy. Figure 1b shows that the van der Waals energy of the model system increased linearly with the increasing solution concentration, which had the opposite trend in electrostatic energy curves. When the protein sample was immersed in the divalent ion solution, the van der Waals interaction increased more quickly with the salt concentration than the case of protein in the monovalent ion solution. Since the magnitude of the decreasing electrostatic energy was much larger than that of the van der Waals energy, while the bonded energies were independent of the salt concentration, the total energy decreased with the increase of the solution concentration.

#### 3.1.2. Structural Analysis

**Figure 2:**
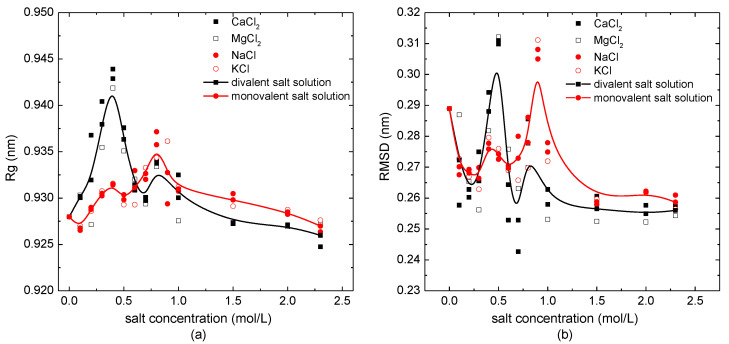
The structural parameters of the protein system versus the external salt concentration. (**a**) The radius of gyration and (**b**) the root mean square deviation.

The salt concentration-dependence of the radius of gyration, *Rg,* and the root mean square deviation (RMSD) are shown in Figure 2a,b, respectively. The solid lines with symbols are the B-spline fit curves based on the average values of structural parameters overall monovalent/divalent salt solutions with different concentrations. As shown in Figure 2a, the values of *Rg* in the case of the protein immersed in salt solutions were mostly larger than those in pure water. The radius of gyration of the protein sample 1BBL changed from 0.925 nm to 0.945 nm in the divalent salt solutions (CaCl_2_, MgCl_2_). Whatever the external salt solution the protein was immersed in, the *Rg* increased to the maximum value and then decreased with the increase of concentration. The maximum value of *Rg* was located at the salt concentration of 0.4 mol/L, when 1BBL was immersed in the salt solutions containing divalent ions. Comparing this to the case of the protein sample in the divalent solution, it presented a small numerical difference of the *Rg* when 1BBL was immersed in the monovalent salt solution (NaCl, KCl). The maximum value of *Rg* was located at 0.8 mol/L, which was double the concentration of the divalent salt solution. It indicates the loose structure of the protein when immersed in the salt solution with a certain concentration.

A similar tendency could also be obtained from the B-spline curves of the RMSD with concentrations in Figure 2b. It presented two visible peak values when the protein sample was in the salt solution with the concentrations of 0.4 mol/L and 0.8 mol/L, which were the same salt concentrations as when the peak value of *Rg* appeared. When the protein was in such concentration of salt solutions, it was not only loose in structure, but it also had a lower stability. In the condition of the protein immersed in the monovalent solutions, the maximum value of the RMSD appeared at 0.8 mol/L, and the second-largest value appeared at 0.4 mol/L. On the contrary, when 1BBL was immersed in the divalent salt solutions, the maximum and second maximum value of the RMSD appeared at 0.4 mol/L and 0.8 mol/L, respectively. With the same result as the concentration dependency of *Rg* shown in Figure 2a, the concentration corresponding to the maximum value of the RMSD in monovalent salt solution was double the one of the divalent salt solution. Except for the protein sample 1BBL in the certain concentration of about 0.4 mol/L or 0.8 mol/L, most of the values of the RMSD were lower than those of the protein sample in a pure water environment. It indicates that the structural stability of the protein sample was slightly increased when it was immersed in the salt solution, except for when the salt concentration was around 0.4 mol/L or 0.8 mol/L. The loose structure with a relatively low stability of this single peptide chain may be relevant, with the maximum solubility of the protein solution at a specific concentration. It helps us to understand the salt dissolution and salting out of the protein from the perspective of the structural analysis.

**Figure 3:**
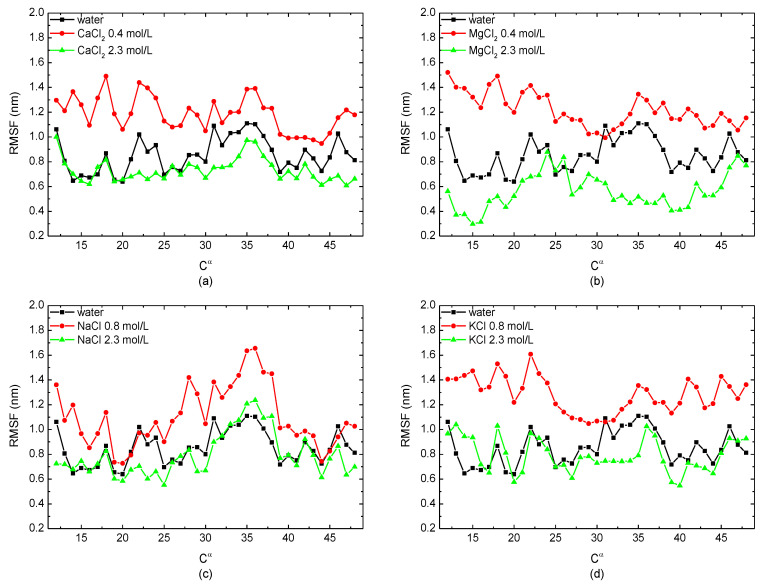
The root mean square fluctuation of the skeleton *C^α^* atom of the protein 1BBL immersed in (**a**) CaCl_2_, (**b**) MgCl_2_, (**c**) NaCl, and (**d**) KCl solutions, respectively.

To study the freedom degree of the skeleton *C^α^* atom along the residue sequence, the root mean square fluctuations (RMSFs) of the protein sample immersed in monovalent/divalent solutions with different concentrations were calculated. In Figure 3, the certain salt concentrations corresponding to the peak value of *Rg* and the RMSD, i.e., 0.4 mol/L in the case of divalent CaCl_2_/MgCl_2_ solutions and 0.8 mol/L in the case of monovalent NaCl/KCl solutions, were selected to compare with the simulation condition of 2.3 mol/L. The curves showed that the RMSF of the protein sample in the salt solutions with a low concentration was higher than the protein in pure water. However, when the salt concentration was high enough, the value of the RMSF was lower or close to that when the protein was in a no-salt environment. It demonstrated that the protein sample had a higher degree of freedom in low-concentration solutions, but it became more stable in salt solutions with a high concentration. This conclusion is also consistent with the results presented in Figure 2.

### 3.2. Effect of Temperature

#### 3.2.1. Energy Analysis

**Figure 4:**
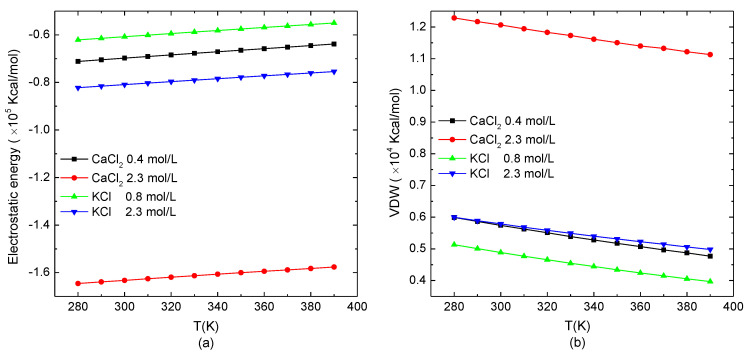
The non-bonded interactions of the protein system versus the temperature. (**a**) Electrostatic potential energy and (**b**) van der Waals energy.

The temperature-dependence of the electrostatic potential energy and van der Waals energy of the protein system in the typical salt solution when the protein presented the loose structure (0.8 mol/L KCl and 0.4 mol/L CaCl_2_) or stable structure in a high salt concentration (2.3 mol/L KCl/CaCl_2_) is shown in Figure 4. The temperature range was set from 280 K to 390 K. As shown in Figure 4a, the electrostatic potential energy of the system increased linearly with the increase of the temperature. Different from Figure 1a, in which the slope of the electrostatic potential energy as a function of the salt concentration was related to the type of ions in the salt solutions, the slope of the electrostatic potential energy with the temperature was independent of the salt solutions. Figure 4b shows that the van der Waals energy of the simulation system decreased linearly with the increase of the temperature. Compared to the effect of the salt concentration, the temperature showed the reverse effect on the protein. Meanwhile, the changes of the electrostatic potential energy and van der Waals energy as functions of temperature were opposite. Since the energy range of increasing the electrostatic potential energy with temperature was much larger than the one of decreasing van der Waals energy, the total energy of the system had an increasing tendency with the environmental temperature. It demonstrates that the increase of the temperature intensified the violent degree of the movement of atoms in the protein molecular. Thereby, the electrostatic potential energy was increased, and the van der Waals energy was reduced with the relatively increasing distance between the charges and the atoms in the protein system.

#### 3.2.2. Structural Analysis

**Figure 5:**
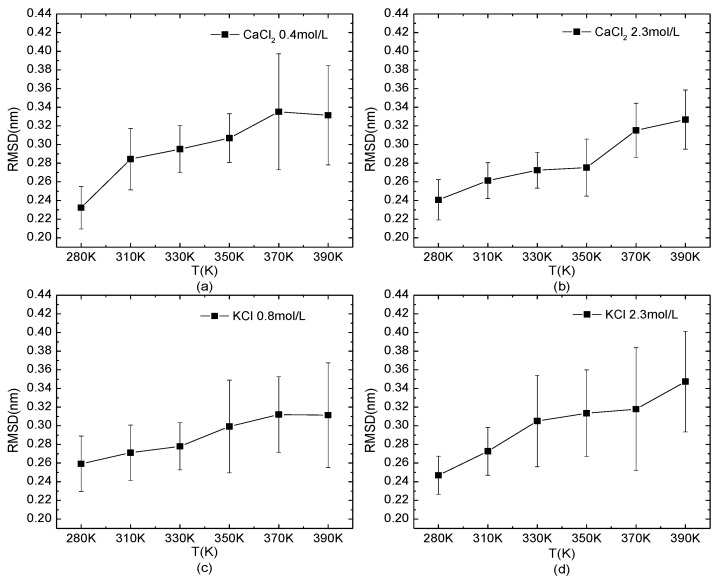
The root mean square deviation of the protein system versus the temperature. The protein 1BBL was immersed in (**a**) 0.4 mol/L CaCl_2_, (**b**) 2.3 mol/L CaCl_2_, (**c**) 0.8 mol/L KCl, and (**d**) 2.3 mol/L KCl solutions, respectively.

The temperature-dependence of the RMSD of the protein 1BBL in the salt solutions is shown in Figure 5. The typical salt concentrations at which the protein presents the loose structure (0.4 mol/L CaCl_2_ and 0.8 mol/L KCl) or stable one (2.3 mol/L KCl/CaCl_2_) were chosen as the same environmental condition in Figure 4. The curves of the RMSD as a function of temperature showed an increased value and fluctuation, indicating the decreased structural stability when the protein was under high-temperature conditions. Compared to the effect of the salt concentration on the protein, the influence of temperature was more intuitive on the structural stability of the protein.

**Figure 6:**
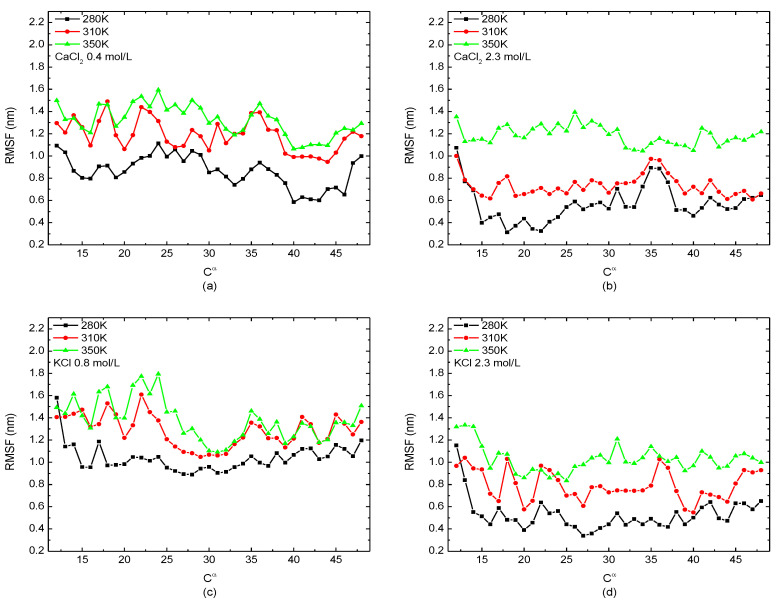
The root mean square fluctuation of the skeleton *C^α^* atom of the protein 1BBL immersed in (**a**) 0.4 mol/L CaCl_2_, (**b**) 2.3 mol/L CaCl_2_, (**c**) 0.8 mol/L KClm and (**d**) 2.3 mol/L KCl solutions, respectively.

The relationship between the value of the RMSF of the backbone *C^α^* atom among protein molecular and certain temperatures (280 K, 310 K, and 350 K) with different salt solutions (0.4 mol/L CaCl_2_, 0.8 mol/L KCl, and 2.3 mol/L CaCl_2_/KCl) is presented in Figure 6. As the same tendency shown in Figure 5, the protein was more unstable in the high-temperature solution as the value of the RMSF increased with the temperature at any salt concentration, indicating the degree of freedom of the skeleton $C^{\alpha }$ atoms increases with the temperature.

## 4. Conclusions

In this article, the effect of temperature, the salt solution with different valences, and salt concentration on the structural characteristics of the protein were investigated by MD simulations. The analysis of the simulation results revealed several findings: (1) The change of the electrostatic potential energy is always opposite to van der Waals energy of the protein system, and the alternating quantity of the electrostatic potential energy is much larger than the one of van der Waals energy with the variation of salt concentration or temperature. (2) With the increasing concentration of salt solutions, the values of the RMSD and *Rg* both increase first and then decrease. These two structural parameters reach the maximum value when 1BBL is immersed in a monovalent ion solution with a concentration of about 0.8 mol/L, which is double the concentration in the case of 1BBL immersed in divalent solutions. It indicates that the structure of the protein chain at this certain concentration is the loosest and that the stability is poor. This conclusion was also obtained from the analysis of the RMSF. (3) The values of the RMSD and RMSF both increase with the increase of temperature, and the fluctuation of the RMSD is more obvious at a high temperature. It shows that the stability of the protein decreases as the atom kinetic energy increases. This research on the protein immersed in salt solutions by MD simulations can provide an atomistic-level description of the protein. These conclusions offer a theoretical basis for understanding the influence of the external environment on the structural transformation of the protein. It could promote the development in both functional genomics and biotechnology.

## Data Availability

Data is contained within the article.
